# Analysis of the characteristics of optical coherence tomography angiography for retinal cavernous hemangioma: A case report

**DOI:** 10.1097/MD.0000000000009940

**Published:** 2018-02-16

**Authors:** Shuyuan Lyu, Ming Zhang, Ruikang K Wang, Yunxia Gao, Qinqin Zhang, Xiaoxue Min

**Affiliations:** a Department of Ophthalmology, West China Hospital of Sichuan University, Wuhou District, Chengdu, Sichuan, China; b Department of Bioengineering, University of Washington, Seattle, Washington, USA.

**Keywords:** cavernous hemangioma, OCTA, optical coherence tomography angiography, retinal cavernous hemangioma, retinal hamartoma, retinal neoplasms

## Abstract

**Rationale:**

Retinal cavernous hemangioma is a rare congenital vascular malformation with typical fundus changes. Optical coherence tomography angiography (OCTA), which is in rise in the recent years, is a rapid and noninvasive technology to assist in obtaining information regarding the blood flow changes in the fundus lesions from different layers without injecting a contrast agent.

**Patient concerns:**

A 40-year-old male patient with visual occlusion in the right eye for >1 month was reported.

**Diagnoses:**

Retinal cavernous hemangioma was diagnosed by fundus examination, fluorescein angiography (FA) and OCTA, and the characteristics of OCTA images were analyzed.

**Interventions:**

The lesion occurred outside the macula, the central vision remained basically normal, and no significant complications were noted in this patient. Therefore, we preferred to regularly follow-up without therapeutic intervention.

**Conclusions:**

OCTA can display fundus blood flow and vascular lesions noninvasively and rapidly. On OCTA, retinal cavernous hemangiomas showed characteristic changes and have good correspondence with fundus imaging and FA examinations. Moreover, OCTA remains more sensitive to vascular abnormalities, and imaging remains clearer, providing new diagnosis and follow-up route for this disease.

## 1 Background

Retinal cavernous hemangioma is a rare retinal hamartoma.^[1]^ Retinal cavernous hemangioma is commonly seen in adolescents^[2]^ and the eyes involved with this condition are mostly monocular, accompanied by cavernous hemangiomas in the skin and central nervous system.^[2–8]^ Optical coherence tomography angiography (OCTA), which is in rise in the recent years, is a rapid and noninvasive technology. This assists in obtaining information regarding the blood flow changes in the fundus lesions from different layers such as the retina, choroid, and so on without injecting a contrast agent. OCTA is a noninvasive diagnostic approach for the occurrence and development of various fundus diseases from new perspectives and has become a hot research topic.^[8,9]^ OCTA image characteristics of retinal cavernous hemangioma are rarely reported, and OCTA remains a new means of examination. Therefore, in our study, we used OCTA scanning for 1 patient who has been diagnosed with retinal cavernous hemangioma. The characteristics of OCTA images were analyzed and were reported as follows.

## 2 Case report

A 40-year-old male patient was first treated in our hospital with visual occlusion in the right eye for >1 month. The patient did not complain any other discomforts, and there was no positive finding in medical history. Eye examination showed best corrected visual acuity to be 20/25 in the right eye and 20/20 in the left eye. Anterior segment examination of both the eyes and fundus examination of the left eye showed no other obvious abnormalities. Institutional ethics review board policy is not required for observational case reports that do not alter the patient management. Written informed consent was obtained from the patient for the publication.

**Figure 1:**
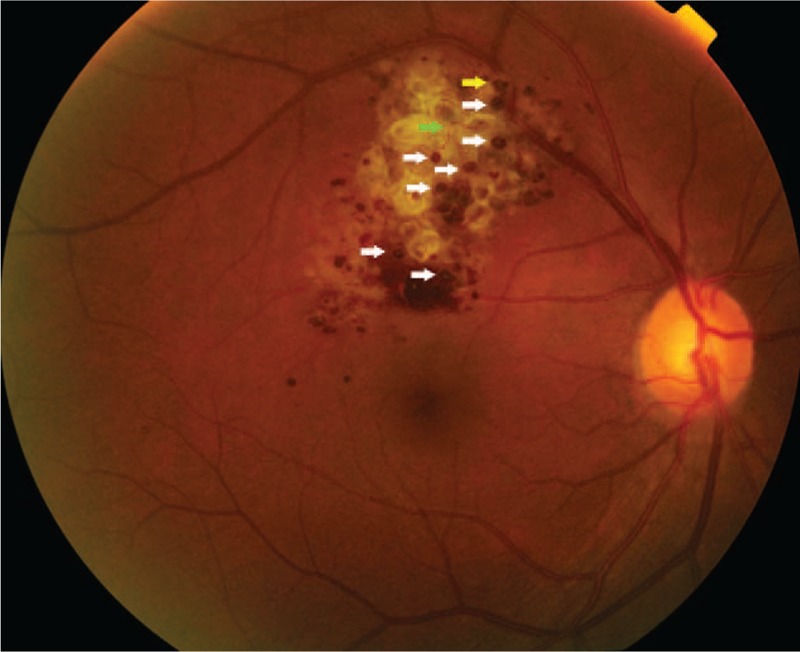
Fundus image of the right eye showing a mass-like yellowish white heterogeneous lesion, which is grape-like bumpy in appearance and protruding from the surface of the retina. Cyst-like changes of partially abnormal dilated vessels are filled with dark red venous blood (white arrow). Liquid level is observed at 1 place (yellow arrow). The retinal blood vessels extending into the tumor from the branches of superotemporal vascular arcade are widened and dilated (green arrow).

Fundus imagine (Topcon, Tokyo, Japan) of the right eye showed a mass-like yellowish white heterogeneous lesion of 1.5 papillary diameter above the temporal region of optic papillary. Part of the lesion was under superotemporal vascular arcade, which is grape-like bumpy in appearance and protruding from the surface of the retina (Fig. 1). Cyst-like changes of partially abnormal dilated vessels were visible and were filled with dark red venous blood (Fig. 1, white arrow). Moreover, liquid level was observed at 1 place (Fig. 1, yellow arrow). The retinal blood vessels extending into the tumor from the branches of superotemporal vascular arcade are widened and dilated (Fig. 1, green arrow). Surface of the tumor was covered by a large number of yellowish white heterogeneous fibrous tissues. Morphology of retinal blood vessels around the lesion was basically normal, and local hemorrhage at the lower part of the lesion was observed. Moreover, petechial hemorrhage was scattered around the lesion, and there was no obvious lipid exudation.

**Figure 2:**
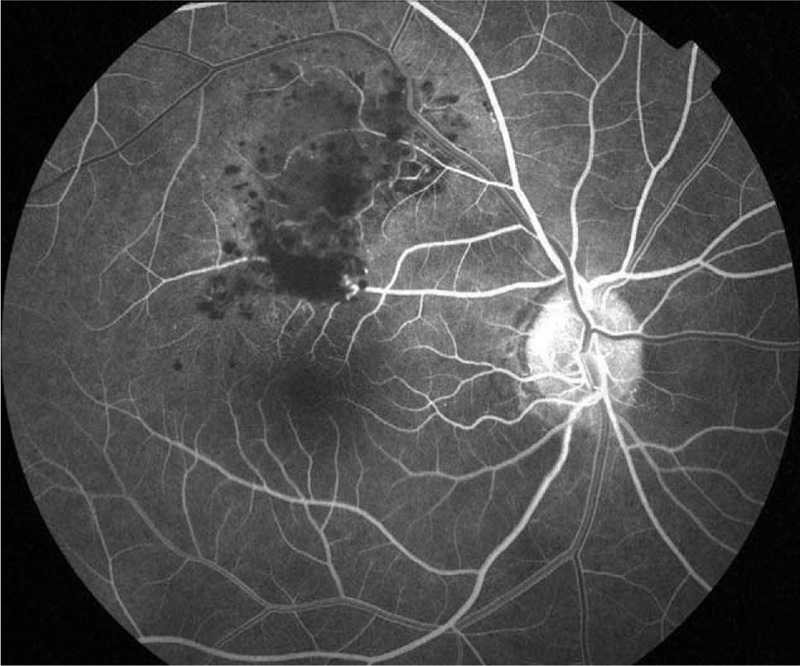
FA in the early stage indicates that fluorescein filling is slow with hypofluorescence. FA = fluorescein angiography.

**Figure 3:**
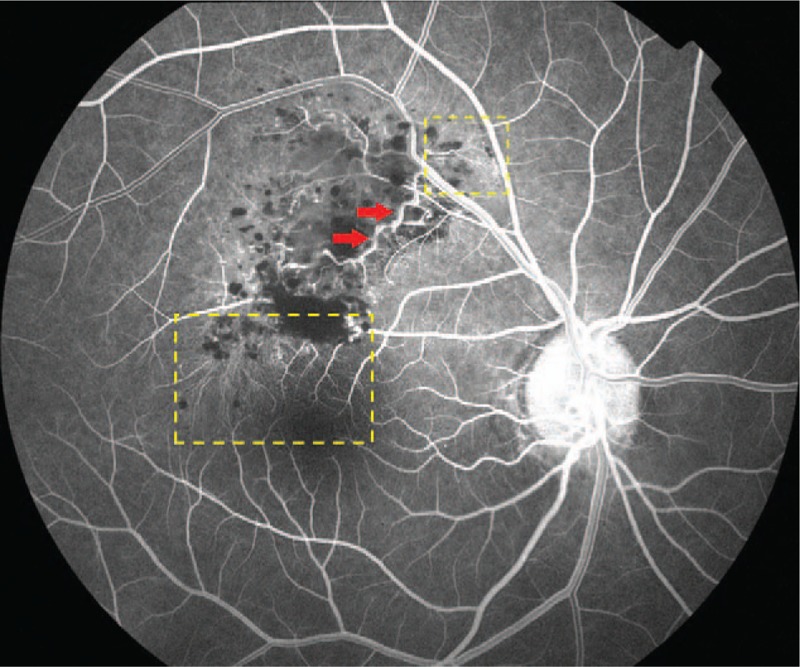
FA on laminar flow in the veins. Retinal angioplerosis stretched into the tumor from superotemporal vascular arcade (red arrow). This in turn has widened and irregularly expanded the blood vessels in root-like shape, while the ends appears in brush-like shape (yellow dotted box). FA = fluorescein angiography.

**Figure 4:**
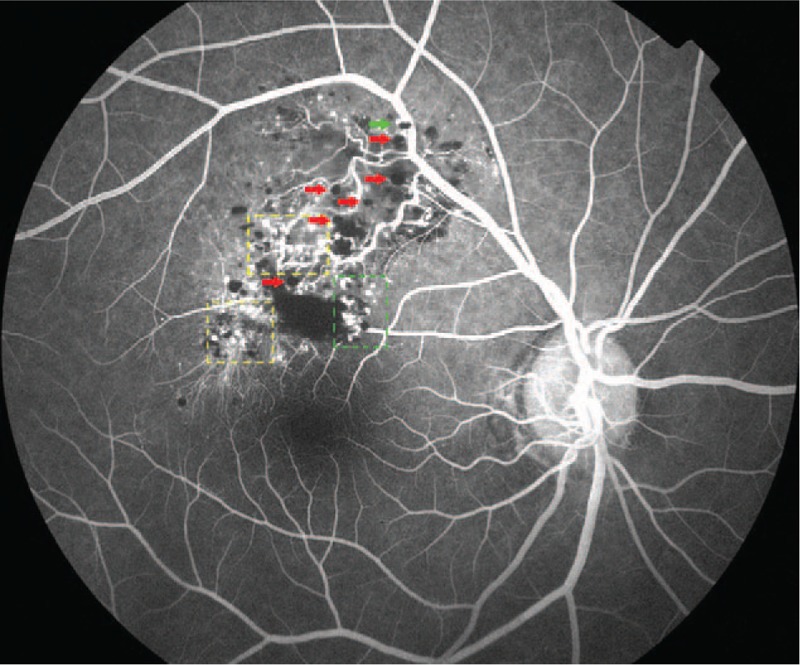
FA in the venous phase, the fluorescein in the tumor is gradually filled and appears in flake-like, grape-cluster-like (yellow dotted box), and cap-like clusters with strong fluorescence (green dotted box). Quasi-circular hypofluorescence (red arrow) and semicircular liquid level (green arrow) are observed. FA = fluorescein angiography.

**Figure 5:**
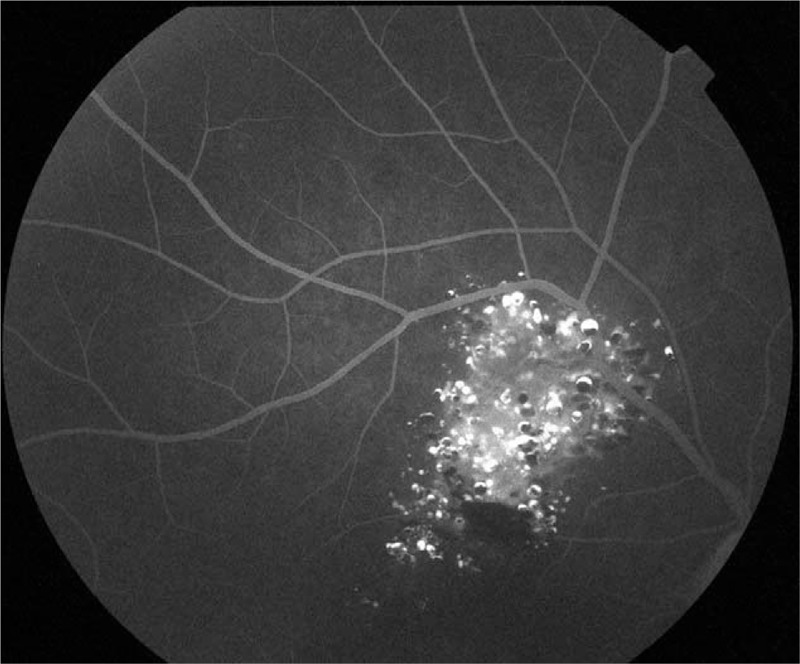
No obvious fluorescein leakage was noted in the late stage of FA. FA = fluorescein angiography.

Fluorescein angiography (FA, Topcon, Tokyo, Japan) indicated that fluorescein filling in the early stage was slow with hypofluorescence (Fig. 2), and retinal angioplerosis stretching into the tumor from superotemporal vascular arcade was visible (Fig. 3, red arrow) on laminar flow in the veins (Fig. 3). This in turn widened and irregularly expanded the blood vessels in root-like shape, while the ends appeared in brush-like shape (Fig. 3, yellow dotted box). Until the venous phase (Fig. 4), the fluorescein in the tumor was gradually filled and appeared in flake-like, grape-cluster-like (Fig. 4, yellow dotted box), and cap-like cluster with strong fluorescence (Fig. 4, green dotted box). Quasi-circular hypofluorescence (Fig. 4, red arrow) and semicircular liquid level (Fig. 4, green arrow) were observed locally. No obvious fluorescein leakage was noted in the late stage (Fig. 5) of FA. Fluorescence was covered by scattered hemorrhage locally at the lower part of the lesion and around the lesion.

**Figure 6:**
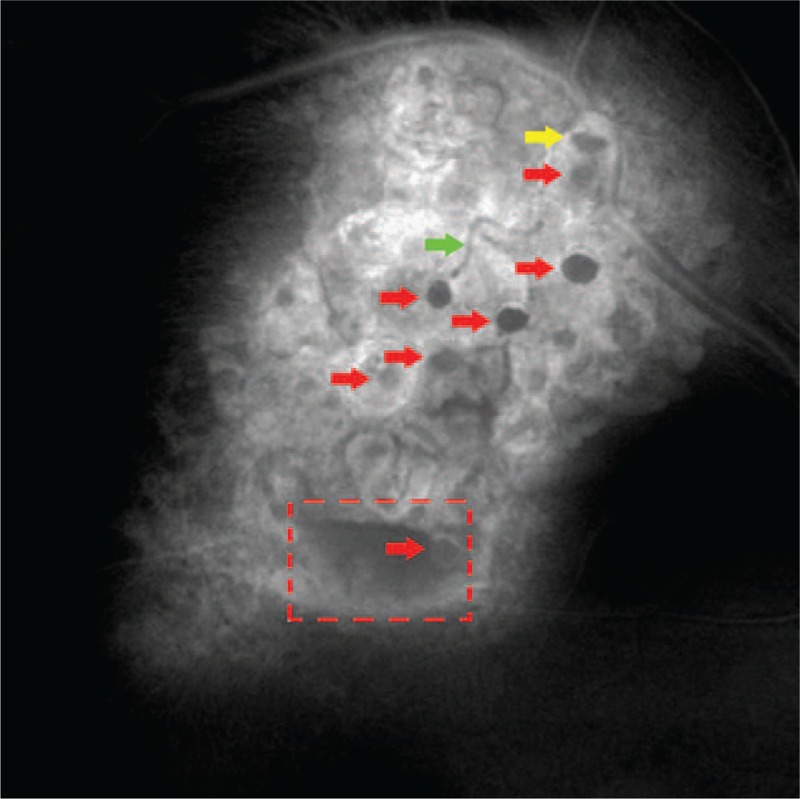
Enface image, the surface of the lesion remains uneven with moderately reflective grape-cluster-like nodular changes (red arrow), and liquid level is visible (yellow arrow). The branch vessels penetrating into the lesion is tortuous and low reflective (green arrow). A boat-like low reflective area (red dotted box) corresponds to the hemorrhage parts on the fundus image.

On Enface (Fig. 6, CIRRUS; Carl Zeiss Meditec, Inc, Dublin, CA), the whole lesion showed heterogeneous medium to highly reflective clumps, and the surface of the lesion remained uneven with moderately reflective grape-cluster-like nodular changes (Fig. 6, red arrow). Among them, several round low reflective shadows corresponding to cyst-like abnormal vessels filled with dark red venous blood appeared on the fundus images and liquid level was also visible (Fig. 6, yellow arrow). Superotemporal vascular arcade showed moderate reflection on the Enface image. The branch vessels penetrating into the lesion was tortuous and low reflective (Fig. 6, green arrow), which were adjacent to the low reflective cyst-like changes that are visible locally. Local glial fibrosis showed unevenly high reflection and a boat-like low reflective area (Fig. 6, red dotted box) in the lesion (hemorrhage parts on the fundus images).

**Figure 7:**
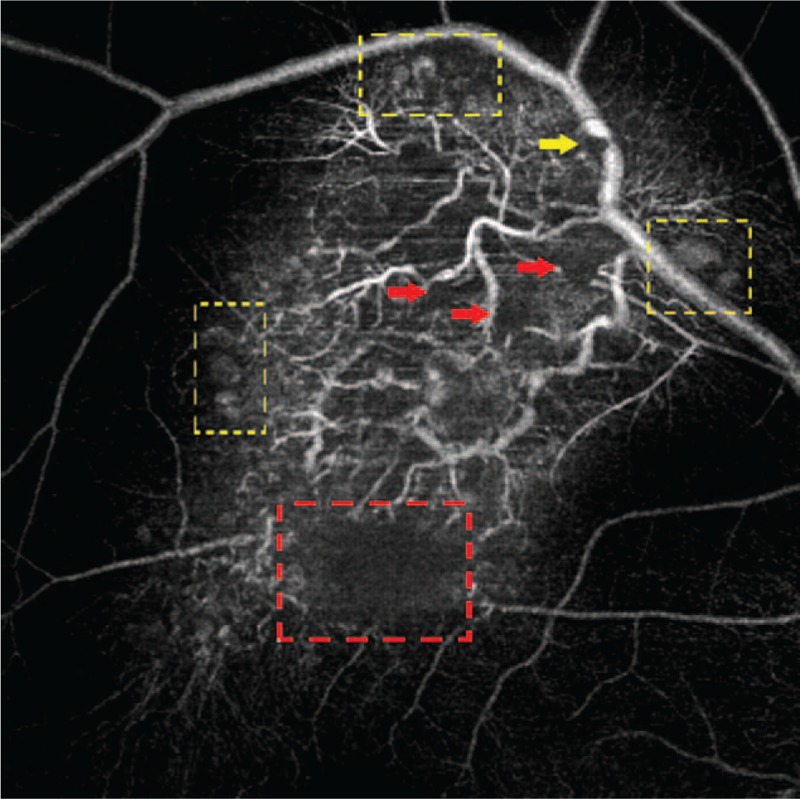
OCTA image showing nodular and grape-like cluster changes on the margin of the lesion (yellow dotted box). Some of the circular low reflective areas correspond to the low reflective areas on the Enface (red arrow) and liquid level is also visible (yellow arrow). Boat-like area is also visible (red dotted box). OCTA = optical coherence tomography angiography.

On OCTA (Fig. 7, CIRRUS Carl Zeiss Meditec, Inc, Dublin, CA), nodular and grape-like cluster changes were observed on the margin of the lesion (Fig. 7, yellow dotted box), which were moderately reflective. Superotemporal vessels and vessels inside the lesion were highly reflective. Retinal vessels tortuously expanded into the lesion from the branches of superotemporal vascular arcade, which were in root-like shape in the end and brush-like shape for small branches. Furthermore, circular low reflective areas can be observed. Some of these covered the retinal vessels below them, corresponding to the low reflective areas on the Enface (Fig. 7, red arrow), and liquid level was also visible (Fig. 7, yellow arrow). Boat-like area in the lesion below them also corresponded to Enface (Fig. 7, red dotted box).

Because the lesion occurred outside the macula, the central vision remained basically normal and no significant complications were noted in this patient. Therefore, we preferred to regularly follow-up without therapeutic intervention.

## 3 Discussion

Retinal cavernous hemangioma is a rare congenital vascular malformation that is inherited in an autosomal-dominant manner.^[1,2,4,8]^ This is commonly seen in adolescents mostly in females compared to males. Moreover, the eyes involved with the condition are mostly monocular.^[2]^ Some patients have diseases combined with intracranial and skin cavernous hemangiomas and are known as neuro-ocular skin syndrome.^[2–8]^ Under an ophthalmoscope, the features of fundus are polycystic, dark red, and purple grape-like tumors of different sizes accumulating together. The cystic wall of the tumor was thin, and part of the surface was covered by a white fiber membrane, located in the inner layer of the retina, and also on the surface of the optic papillary that is slightly bulged in shape. Liquid level with plasma separated from the blood cells was sometimes visible inside the cystic cavity, and a small amount of hemorrhage was occasionally visible subretinally or inside the vitreous body, disappearing automatically. There was no lipid exudation in the retina around the tumor. Pathways and diameters of retinal arteries and veins appeared normal.^[1,2,10–13]^ If hemangioma occurred outside the macula, the central vision remained basically normal (such as the patient in our study), otherwise central vision may be affected.^[2,5,14]^

FA examination of this disease was extremely special, which remained of significant importance for diagnosing.^[2,4,6,12]^ In the early stage of FA, fluorescence filling inside the tumor was very slow and incomplete, showing hypofluorescence. The fluorescence slowly advanced to the center from the periphery of the tumor. Until advanced stage of FA, flake-like hyperfluorescence spots can be observed inside some cysts, and the time of fluorescence vanishing was relatively long, showing a characteristics of “cap-like fluorescence.” This is mainly caused by the separation of plasma and blood cells inside the cyst as detected by the ophthalmoscope. Plasma above the liquid level showed hyperfluorescence, while fluorescence below was covered by deposited blood cells. This characteristic fluorescence image suggests that there was a relative independence between the blood flow inside the tumor and blood flow of the retina. A rare fluorescein leakage during the entire process of FA was observed.^[6]^

OCTA is a noninvasive diagnostic approach used to test the rapid blood flow of the fundus and can better display the blood flow in fundus lesions.^[8,9]^ OCTA image of our case showed multiple nodular and grape-cluster-like changes in the lesion area, which were moderately reflective and obvious in the periphery of the lesion. These in turn corresponded to the typical grape-cluster-like changes by fundus examination, FA, and Enface. Therefore, it was speculated that such changes were correlated with abnormal vascular dilation of the cystic cavity. Moreover, OCTA image was less affected by glial fibrosis compared with other imaging examinations, and it can display a single nodular change more clearly than other imaging approaches. Thus, we believed that OCTA had better sensitivity in displaying this disease. The scattered circular low reflective shadows in the lesion corresponded to the purple–red circular lesions in the fundus image, circular hypofluorescence in FA, and circular low reflective shadows in Enface scanning. These are cystic cavities formed by abnormal expansion of the vessels. A dark red venous blood-filled cystic cavity was observed, and hence was purple–red on fundus image. However, it formed shadows in FA, Enface, and OCTA due to dense coverage of the blood cells and extremely slow blood flow.^[6,12]^ Liquid level can be observed in cyst-like changes locally in the lesion. A typical “cap-like fluorescence” on FA was observed. It was highly reflective on the upper layer and low reflective on the lower layer on Enface and OCTA images. Due to relatively stagnant blood flow within the tumor and separation of the plasma and blood cells, blood cells were deposited in the lower layer due to gravity, while the plasma floated on the upper layer as it was lighter.^[2,6,12]^ When imaging, plasma in the upper layer was stained, which was highly reflective on Enface and OCTA, and blood cells in the lower layer were deposited to cover the fluorescence due to low reflection on Enface and OCTA, thus forming typical “cap-like” changes. The mechanisms for the formation of above nodular lesions, grape-cluster-like lesions, circular low-reflective lesions, and “cap-like” changes were similar, and were all related to the formation of a cystic cavity by characteristic abnormal expansion of vessels in the hemangiomas. But the contents in the cystic cavity were different, thereby forming the above differences in imaging performances. Moreover, because the blood composition in the cystic cavity of these abnormally expanded vessels was different from normal retinal vessels, blood flow was relatively stagnant and greatly slowed down. Thus, there was different reflection intensity on OCTA compared to normal retinal vessels, suggesting a relative independence between the blood flow within the tumor and normal retinal blood flow. The above 3 OCTA changes corresponded well with the typical changes of the disease on fundus imaging and FA, thus considered as the characteristic changes of the disease on OCTA. Furthermore, OCTA can extract lesions from specific retinal vascular layer to display the vascular lesions, excluding the shielding effect of surrounding tissues as far as possible. Therefore, we believed that OCTA images were more distinguishable for diseases characterized by this kind of vascular abnormalities.

Although tortuous retinal vessels are not specific changes of this disease, these can also be used as reminders of the disease to some extent. These were better than the package and subsequently cover the effects of glial fibrosis on the fundus and FA images in this case. However, some abnormal vessels were not clearly displayed, and a small amount of thicker abnormal branching vessels located in the superficial area of the lesion were visible on Enface images. However, abnormal vessels located deeply in the lesion or small vessels or vessels wrapped by glial tissues cannot be observed. The relationship of abnormal branching vessels with cystic changes and retinal macrovessels was unclear. Moreover, morphology of the end of the abnormal vessels cannot be displayed. It was observed from OCTA that a number of tortuous retinal vessels expanding into the lesion in this case were from the branches of superior temporal retinal vessels, which formed root-like and brush-like changes at the end. Moreover, the vessels were intertwined with lesions of cystic expansion. Retinal vessels surrounding the lesion were relatively normal, further demonstrating that OCTA was advantageous in displaying vascular lesions over others.

## 4 Conclusions

OCTA displays fundus blood flow and vascular lesions noninvasively and rapidly. On OCTA, cavernous hemangiomas showed characteristic changes and have good correspondence with fundus imaging and FA examinations. Moreover, OCTA can extract specific retinal layer to perform stratified display of the vascular lesions, excluding the shielding effect of the surrounding tissues as far as possible. Therefore, OCTA remains more sensitive to vascular abnormalities, and imaging remains clearer, providing new diagnosis and follow-up route for this disease. Till date, there are few studies reported on this disease, and more studies are needed to analyze the characteristics of the disease with OCTA. This helps to further confirm the sensitivity and specificity of OCTA as a diagnostic strategy for this disease.
